# Magnetite-Based Nanostructured Coatings Functionalized with *Nigella sativa* and Dicloxacillin for Improved Wound Dressings

**DOI:** 10.3390/antibiotics12010059

**Published:** 2022-12-29

**Authors:** Gabriela Dorcioman, Ariana Hudiță, Bianca Gălățeanu, Doina Craciun, Ionel Mercioniu, Ovidiu Cristian Oprea, Irina Neguț, Valentina Grumezescu, Alexandru Mihai Grumezescu, Lia Mara Dițu, Alina Maria Holban

**Affiliations:** 1 Lasers Department, National Institute for Lasers, Plasma and Radiation Physics, 409 Atomistilor Street, 077125 Magurele, Romania; 2 Department of Biochemistry and Molecular Biology, University of Bucharest, 050095 Bucharest, Romania; 3 National Institute of Materials Physics, 405A Atomistilor Street, 077125 Magurele, Romania; 4 Department of Inorganic Chemistry, Physical Chemistry and Electrochemistry, Faculty of Applied Chemistry and Materials Science, Politehnica University of Bucharest, 1-7 Gheorghe Polizu Street, 011061 Bucharest, Romania; 5 Academy of Romanian Scientists, Ilfov No. 3, 050044 Bucharest, Romania; 6 Department of Science and Engineering of Oxide Materials and Nanomaterials, Politehnica University of Bucharest, 011061 Bucharest, Romania; 7 Research Institute of the University of Bucharest—ICUB, University of Bucharest, 050657 Bucharest, Romania; 8 Department of Microbiology and Immunology, Faculty of Biology, University of Bucharest, 91-95 Splaiul Independentei Street, 077206 Bucharest, Romania

**Keywords:** magnetite nanoparticles, coatings, drug release, antimicrobial potential, wound dressings

## Abstract

In this study, we report the performance improvement of wound dressings by covering them with magnetite-based nanostructured coatings. The magnetite nanoparticles (Fe_3_O_4_ NPs) were functionalized with *Nigella sativa* (*N. sativa*) powder/essential oil and dicloxacillin and were synthesized as coatings by matrix assisted pulsed laser evaporation (MAPLE). The expected effects of this combination of materials are: (i) to reduce microbial contamination, and (ii) to promote rapid wound healing. The crystalline nature of *core/shell* Fe_3_O_4_ NPs and coatings was determined by X-ray diffraction (XRD). Differential Scanning Calorimetry (DSC) and Thermo Gravimetric Analysis (TGA) have been coupled to investigate the stability and thermal degradation of *core/shell* nanoparticle components. The coatings’ morphology was examined by scanning electron microscopy (SEM). The distribution of chemical elements and functional groups in the resulting coatings was evidenced by Fourier transform infrared (FTIR) spectrometry. In order to simulate the interaction between wound dressings and epithelial tissues and to evaluate the drug release in time, the samples were immersed in simulated body fluid (SBF) and investigated after different durations of time. The antimicrobial effect was evaluated in planktonic (free-floating) and attached (biofilms) bacteria models. The biocompatibility and regenerative properties of the nanostructured coatings were evaluated *in vitro*, at cellular, biochemical, and the molecular level. The obtained results show that magnetite-based nanostructured coatings functionalized with *N. sativa* and dicloxacillin are biocompatible and show an enhanced antimicrobial effect against Gram positive and Gram negative opportunistic bacteria.

## 1. Introduction

Wound management is an important clinical issue worldwide [1,2,3,4,5]. According to the report published by Fortune Business Insights Pvt. Ltd., the global wound care market is projected to grow from 18.51 billion USD in 2022 to 28.23 billion by 2029 [6]. In the whole wound healing process, wound dressings have a crucial role. The main characteristics of an efficient dressing are to reduce the risk of infection, minimize the pain, apply compression, protect the wound from secondary injury, facilitate the removal of excess exudate, and promote better and rapid healing [7,8].

The wound site is a suitable environment for the colonization and proliferation of viruses, bacteria, or fungi. Normally, these pathogens are overtaken and eliminated by white blood cells and other components of the immune system, but there are many cases when the body’s defence mechanism is overcome and chronic infections associated with the formation of (mono-or polymicrobial) biofilms appear [9]. By focusing on microbial cell walls or membranes, cellular respiration processes, or quorum sensing systems, natural substances have demonstrated their effectiveness in relation to the present antibiotic resistance issue [10]. The enhanced hydrophobicity, volatility, lipophilicity, oxidation sensitivity, and lower solubility and stability of natural substances, however, pose a number of challenges despite their enormous promise [11]. Therefore, their cooperation with nanotechnology-based strategies is required to prevent the advancement of microbial diseases. 

Recent developments in the wound care management domain are focused on dressings containing drugs, including antibiotics/antimicrobials, as an excellent solution in speeding up wound healing, protecting against infections, and in tissue regeneration. Progress has been made in obtaining wound dressings with demonstrated antimicrobial efficiency with the help of nanotechnological tools—nanoparticles (NPs) possessing antimicrobial properties and being used as drug carriers. 

The use of metal and metal oxide NPs (including magnetite—Fe_3_O_4_) in combination with antibiotics and/or natural active substances as antimicrobial agents has specifically concentrated on the wound management area [12]. Fe_3_O_4_ NPs were previously functionalised with different antibiotics such as cefepim, streptomycin, and neomycin [13], which have been applied and tested on different infection microorganisms. 

In the case of natural substances, there are several works regarding Fe_3_O_4_ NPs functionalised with natural substances for the use in wound dressing applications. For example, Anghel et al. investigated the efficiency of a novel wound dressing coating containing Fe_3_O_4_ and *Satureja hortensis* essential oil. The wound dressings exhibited improved antimicrobial properties, preventing *Candida albicans* colonization and biofilm development [14]. The aim of a study by Radulescu et al. was to develop a biocompatible and antiinfection coating for wound dressings, containing Fe_3_O_4_ NPs functionalized with patchouli essential oil in order to impart antimicrobial properties to the dressings [15]. In another study by Chircov et al., the research group developed nanostructured systems based on Fe_3_O_4_@SiO_2_ core–shell NPs and three different types of essential oils, i.e., thyme, rosemary, and basil, to be potentially used in wound antimicrobial therapies. The antimicrobial properties of the synthesized nanocomposites were assessed by *in vitro* tests on *Staphylococcus aureus*, *Pseudomonas aeruginosa*, *Escherichia coli*, and *Candida albicans* [16].

These NPs have significant potential for the administration of pharmacological substances, as they can enhance biocompatibility, ensure targeted, controlled, and prolonged release of therapeutic compounds, and reduce the amount of bioactive compounds needed for the therapeutic effect desired in many biomedical applications [17,18,19,20,21]. Fe_3_O_4_ NPs properties (such as high surface area, size and size distribution) are closely related to the synthesis method applied to produce them (ex: co-precipitation [22,23,24], sol-gel [25,26], microemulsion [27,28], sonochemical [29], hydrothermal [30,31], electrochemical [32], thermal decomposition [33], polyol [34,35], and biological synthesis [36,37], etc.). 

The antimicrobial nature of the Fe_3_O_4_ NPs can be obtained by anchoring the therapeutic agent of interest [38,39]. In wound care management, Fe_3_O_4_ NPs, which act as vectors for the active substances, must ensure a slow, continuous drug delivery and release and avoid the evaporation and absorbance of active components in the dressings’ texture [40].

Dicloxacillin sodium monohydrate (DCX), a semisynthetic isoxazolyl penicillin, exhibits antimicrobial activity against a wide variety of Gram-positive bacteria, as well as stability against penicillinases and a low level of toxicity [41]. DCX has shown activity against *Stafiloccocus aureus*, *Streptococcus pyogenes*, *Streptococcus pneumoniae*, *Streptococcus epidermidis*, *Streptococcus viridans*, *Streptococcus agalactiae*, and *Neisseria meningitides* [42,43,44]. It is currently applied with success in the treatment of bacterial infections such as bone, ear, skin, urinary tract infections, and pneumonia [45,46]. 

*Nigella sativa* seeds have been used in many ancient cultures due to their high content of biologically active essential oils to treat skin diseases, gastric and heart conditions, pulmonary illnesses, various infections, diabetic wounds, etc. [47,48,49,50,51]. Nevertheless, seeds/powder (PNS) and oils (NS) of *N. sativa* are well known for their anticancer [52,53], antimicrobial [54,55], anti-inflammatory [56], antioxidant [57], glucose lowering [58,59], antihistaminic [60], immune booster [61,62], antiparasitic [63], and hepatoprotective properties [64,65,66]. The biofilm inhibition effectiveness of NS compounds was proved against various human pathogenic microorganisms. It has been revealed that NS active substances displayed a considerable bactericidal activity against *S. aureus* [51,67,68], *S. epidermidis,* and *Enterococcus faecalis* biofilm growth and development [69]. *N. sativa* essential oil and its active compounds, thymoquinone and carvacrol, modulate antibiotic resistance in *Listeria monocytogenes* against various antimicrobials [70]. Furthermore, we noted the isolation of multi-drug resistant *S. aureus* from diabetic wounds and that more than half of isolates were susceptible to different concentrations of *N. sativa* oil [51].

Ultimately, by merging the above-mentioned ideas, we report on the deposition of nanostructured coatings from Fe_3_O_4_ functionalized with dicloxacillin (DCX) antibiotic and *Nigella Sativa* essential oil (NS) or powder (PNS) by matrix assisted pulsed laser evaporation (MAPLE). The novelty of our work consists in obtaining wound dressing coatings which benefit from the combined effects of both natural substances and antibiotics which are delivered by nanostructured vectors. The scope of our work is to obtain improved wound dressings and to have a double outcome: (i) a reduction in microbial contamination and (ii) the promotion of wound healing.

## 2. Results and Discussions

### 2.1. Physico-Chemical Characterization of Fe_3_O_4_ Core/Shell Nanoparticles 

**Figure 1:**
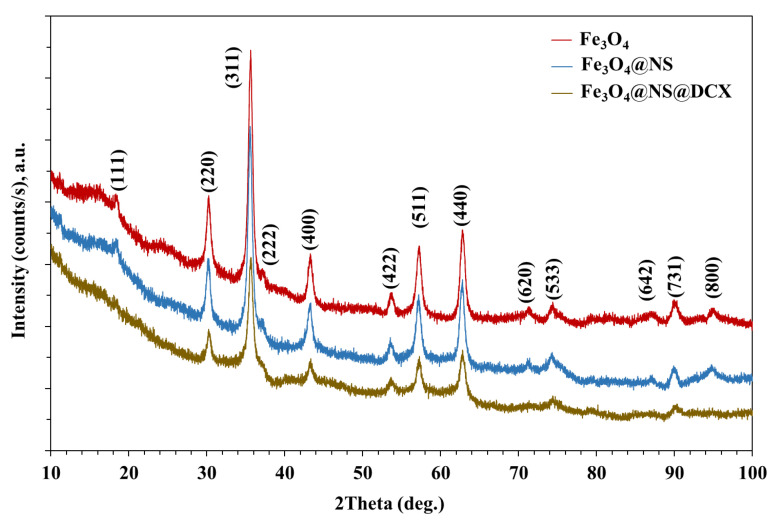
XRD patterns of the Fe_3_O_4_ NPs (red), functionalised with NS oil (blue) and *core/shell* of NS and DCX (brown).

**Figure 2:**
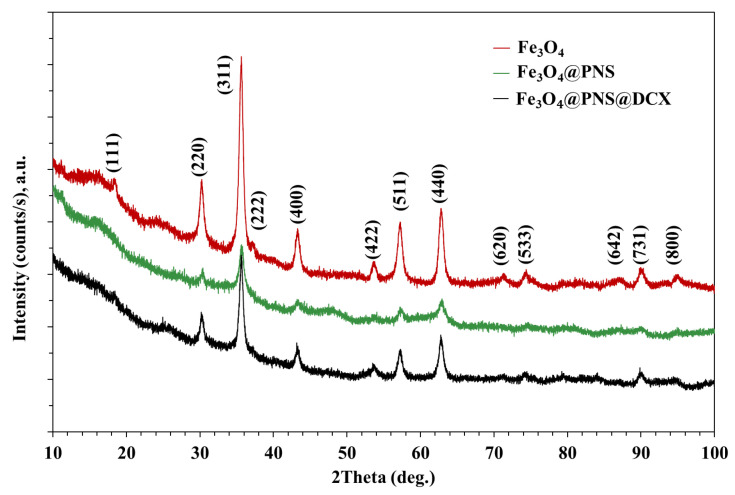
XRD patterns of the Fe_3_O_4_ NPs (red), functionalised with PNS (green) and *core/shell* with NS and DCX (black).

The X-ray difraction (XRD) analysis highlights the presence of strong diffraction interferences that are characteristic of the mineralogical phase, Fe_3_O_4_, according to the ICDD database. The diffraction peaks of synthesized Fe_3_O_4_ NPs (Figure 1 and Figure 2) were detected at 2θ = 18.28°, 30.08°, 35.43°, 37.06°, 43.06°, 53.42°, 56.94°, 62.53°, 70.93°, 73.97°, 86.72°, and 89.61°, which are assigned to the crystal phases of the planes (111), (220), (311), (222), (400), (422), (511), (440), (620), (533), (642), and (731) [71]. The analysed diffraction peaks were matched well with the typical magnetite XRD patterns (JCPDS file no: 00-003-0863), which confirm the crystallographic system of the cubic structure, having the lattice parameter *a* = 8.3958 Å.

**Figure 3:**
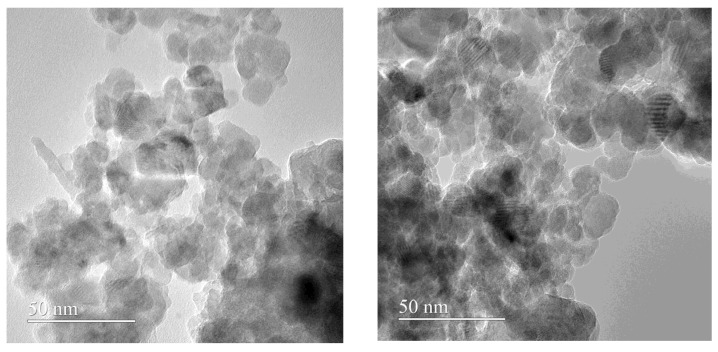
TEM images of the Fe_3_O_4_@PNS@DCX *core/shell* (**left**) and Fe_3_O_4_ @NS@DCX *core/shell* (**right**).

The transmission electron microscopy (TEM) investigation confirmed the nanometric size of our *core shells*, as it is visible from Figure 3. From TEM images the tendency of agglomeration of Fe_3_O_4_ NPs *core/shell* is obvious, together with the presence of individual nanoparticles that possess no preferential morphology and a non-homogenous aspect.

**Figure 4:**
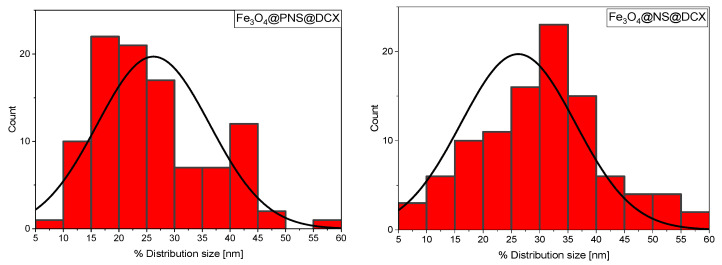
NPs size distribution for Fe_3_O_4_@PNS@DCX (**left**) and Fe_3_O_4_@NS@DCX (**right**) *core-shell*.

In Figure 4 histograms of NPs size distribution for Fe_3_O_4_@PNS@DCX and Fe_3_O_4_@NS@DCX *core/shells* are presented. 

For the Fe_3_O_4_@PNS@DCX *core/shell*, the average size of particulates was determined to be ~26.21 nm, while for Fe_3_O_4_@NS@DCX it was about 30.59 nm.

From a thermo-gravimetric analysis (TGA) the stability and thermal degradation of *core/shell* nanoparticles’ components were evaluated. 

**Figure 5:**
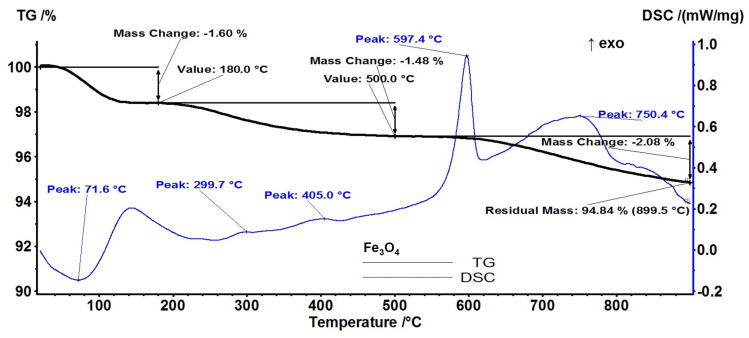
Thermogravimetric analysis of pristine Fe_3_O_4_ NPs.

The pristine Fe_3_O_4_ is losing 1.60% of initial mass up to 180 °C (Figure 5). The process is accompanied by an endothermic effect on the differential scanning calorimetry (DSC) curve, with a minimum at 71.6 °C. This can be attributed to the removing of the water molecules absorbed on the nanoparticles’ surface. In the temperature interval 180–500 °C, the sample is losing 1.48% of initial mass, the corresponding effects being exothermic and very weak at 299.7 and 405 °C. Due to the synthesis method (low temperature, water solvent), the obtained nanoparticles can have a significant amount of -OH moieties on their surface [72]. Therefore, the endothermic effects from -OH groups’ condensation and the H_2_O elimination process can overlap with exothermic effects from the oxidation of magnetite to maghemite or oxidation of the precursor traces [73].

The strong exothermic effect from 597.4 °C represents the transformation of maghemite (γ-Fe_2_O_3_) to hematite (α-Fe_2_O_3_) [74]. The sample is losing 2.08% of initial mass in an oxidative-degradative process between 500–900 °C, the accompanying exothermic effect presenting a broad maximum at 750.4 °C. The residual mass is 94.84%.

**Figure 6:**
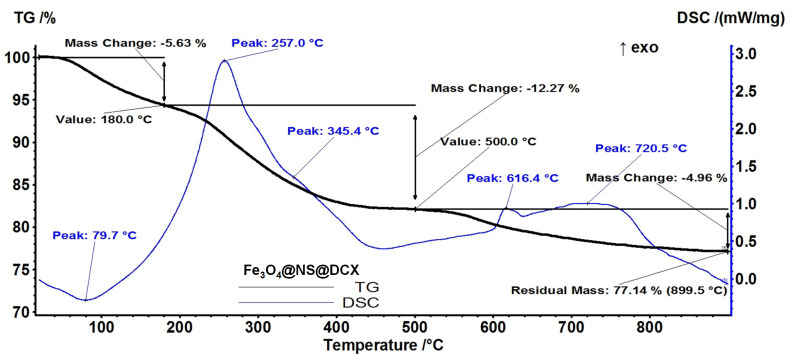
Thermogravimetric analysis of Fe_3_O_4_@NS@DCX.

Regarding Fe_3_O_4_@NS@DCX (Figure 6), the sample is losing 5.63% of the initial mass up to 180 °C. The process is accompanied by an endothermic effect on the DSC curve, with a minimum at 79.7 °C. This can be attributed to the removal of the water molecules absorbed on the nanoparticles’ surface. In the temperature interval 180–500 °C, the sample is losing 12.27% of initial mass in an oxidative-degradative process, which is accompanied by a large, asymmetric exothermic effect on the DSC curve, with a peak at 257 °C and a shoulder at 345.4 °C. This indicates the presence of at least two overlapped oxidative processes. In this step the –OH surface groups are also eliminated.

The exothermic effect from 616.4 °C represents the transformation of γ-Fe_2_O_3_ to α-Fe_2_O_3_. The sample is losing 4.96% of initial mass in an exothermic process between 500–900 °C, the peak on the DSC curve being at 720.5 °C. The residual mass is 77.14%.

**Figure 7:**
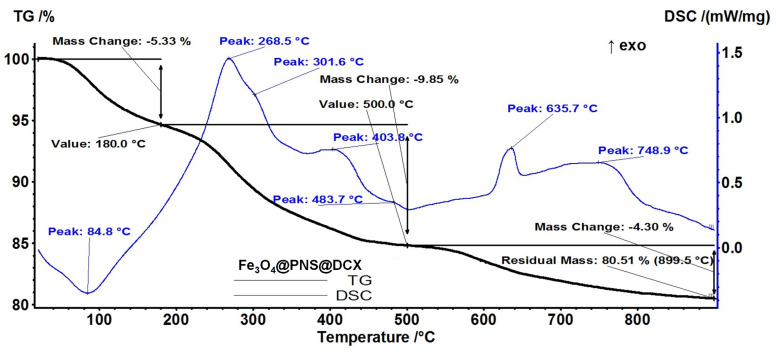
Thermal analysis of Fe_3_O_4_@PNS@DCX.

In the case of Fe_3_O_4_@PNS@DCX (Figure 7), the sample is losing 5.33% of the initial mass in the temperature interval RT-180 °C. The process is accompanied by an endothermic effect on the DSC curve, with a minimum at 84.8 °C, attributed to the removal of the water molecules absorbed on the nanoparticles surface. In the temperature interval 180–500 °C the sample is losing 9.85% of initial mass, in multiple overlapped exothermic processes with peaks at 268.5 and 403.8 °C and shoulders at 301.6 and 483.7 °C. Each of these processes represents an oxidation process, with evolved CO_2_ and H_2_O quantities being larger in the first process. In this step the -OH surface groups are also eliminated.

The exothermic effect from 635.7 °C represents the transformation of γ-Fe_2_O_3_ to α-Fe_2_O_3_. The sample is losing 4.30% of its initial mass in an oxidative-degradative process between 500–900 °C, the accompanying exothermic effect presenting a broad maximum at 748.9 °C. The residual mass is 80.51%.

### 2.2. Physico-Chemical Characterization of Magnetite-Based Nanostructured Coatings

**Figure 8:**
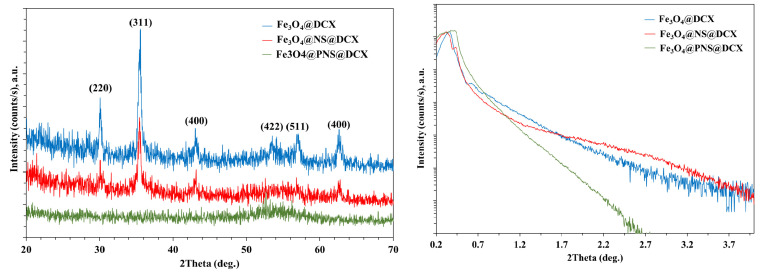
Grazing incidence diffraction pattern in asymmetric geometry with omega = 1.8 (deg) (**left**) and XRR curves (**right**) on DCX functionalized coatings.

From the data acquired by XRD at grazing incidence, on the coatings based on Fe_3_O_4_ functionalized with DCX, a nano-crystalline structure can be distinguished for Fe_3_O_4_@NS@DCX and Fe_3_O_4_@DCX coatings with Fe oxide peaks identified in Figure 6 (blue and red) and an amorphous Fe_3_O_4_@PNS@DCX coatings (green) (Figure 8).

X-ray reflectivity (XRR) analyses revealed that the amorphous coating, Fe_3_O_4_@PNS@DCX, has a higher density than the better crystallized Fe_3_O_4_@NS@DCX and Fe_3_O_4_@DCX coatings.

**Figure 9:**
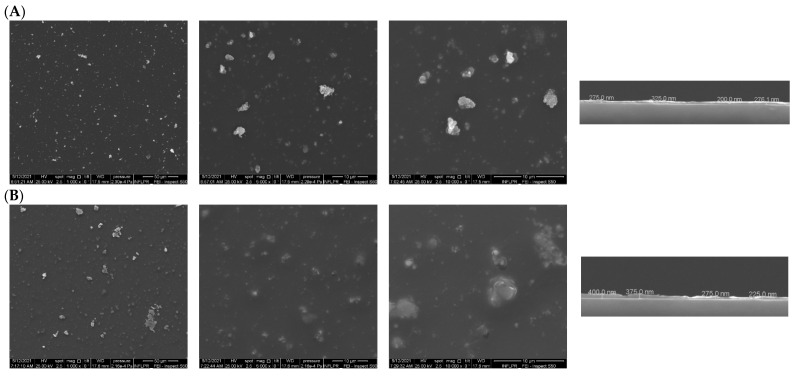
SEM micrographs of coatings deposited by MAPLE technique for (**A**) Fe_3_O_4_@NS@DCX and (**B**) Fe_3_O_4_@PNS@DCX.

Scanning electron microscopy (SEM) micrographs (Figure 9) show the formation of a continuous coating on the substrate, with a surface consisting of isolated, clustered, and randomly scattered nanoparticles. At the same time, it can be observed that the composite coatings present an irregular surface morphology, as further confirmed by the cross-sectional micrographs. The thickness of the coatings containing DCX is in the range of (100–400) nm. In addition, rare local surface anomalies were observed (Figure 9A,B), consisting of clusters of agglomerated nanogranules. The presence of these structures is characteristic of the deposition by pulsed laser technologies [75,76].

**Figure 10:**
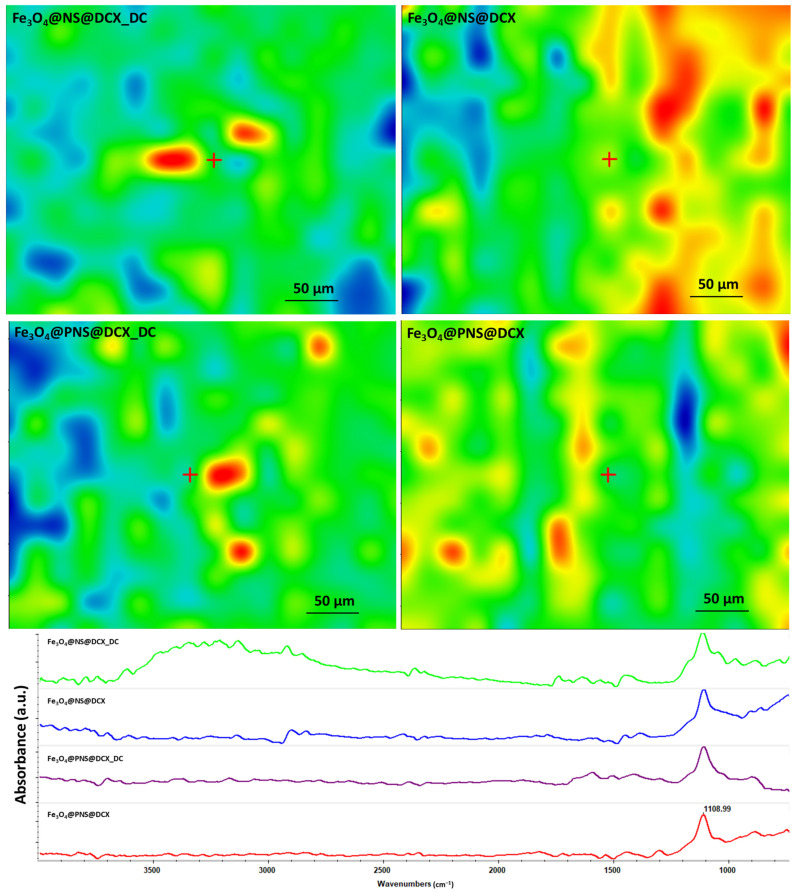
Maps (**above**) and IR spectra (**below**) corresponding drop-cast and coatings obtained at 400 mJ/cm^2^.

In the case of the drop-cast (DC) samples, the existence of predominant blue areas in the complementary IR maps confirms the non-uniformity of the distribution of functional groups on the surface of the Si substrate compared to the coatings with predominant yellow-red areas (Figure 10). However, from the point of view of uniform and efficient transfer of the composite material and the preservation of chemical integrity, optimal results were shown at a laser fluence of 400 mJ/cm^2^.

Compared to drop-cast, laser fluence (400 mJ/cm^2^) did not alter the chemical integrity of the material for all coatings containing DCX. In addition, compared to this for the Fe_3_O_4_@NS@DCX combination, a slight change and reduction of the peaks in the range of 3600–2800 cm^−1^ can be observed. The FT-IR spectra of the composite coatings showed a characteristic band of Fe, attributed to the stretching vibration of Fe bonds with a single O-bond [77], characteristic bands of CH_3_, CH_2_ and carboxyl groups at around ~2900, ~2800 and ~1400 cm^−1^. The FTIR spectra reflected peaks of *NS* through absorption bands at ~3000 and ~2800 cm^−1^ representing O-H and C-H polyphenol stretching. These results are in agreement with previous reports [78].

**Figure 11:**
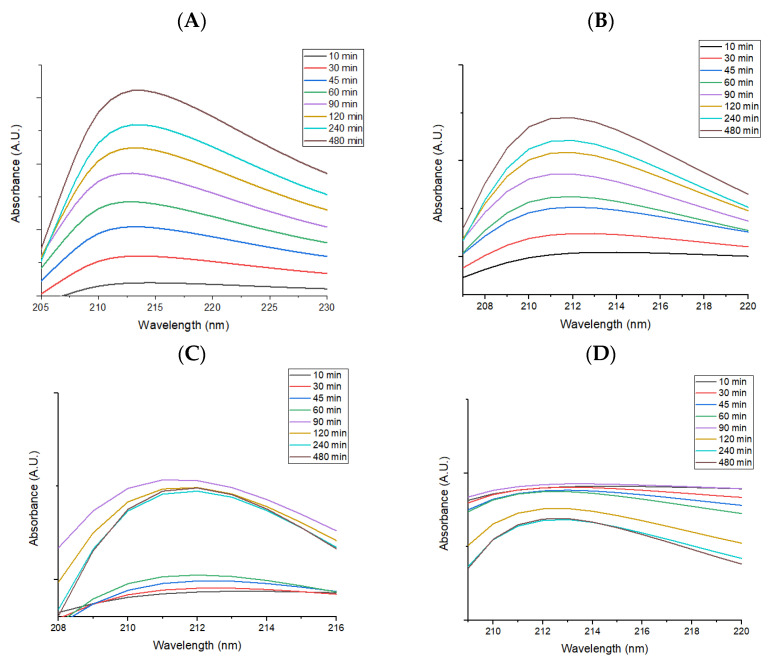
Graphical representation of drug release as a function of time for (**A**) DCX, (**B**) Figure 3. O_4_@DCX, (**C**) Fe_3_O_4_@NS@DCX, and (**D**) Fe_3_O_4_@PNS@DCX coatings.

From the graphical representation of the drug release as a function of the time, it can be seen that the drug is released in the first 10 min after immersion, and continues throughout the tested interval in the case of all studied recipes (Figure 11A–D).

It can be concluded that a significant amount of drug released during the 8 h of testing destroys the adherent microbial strains, while a smaller amount of drug released in the following days prevents the formation of bacterial biofilm that can cause infections.

### 2.3. Biological Evaluation of Magnetite-Based Nanostructured Coatings

#### 2.3.1. Antimicrobial Effect

**Figure 12:**
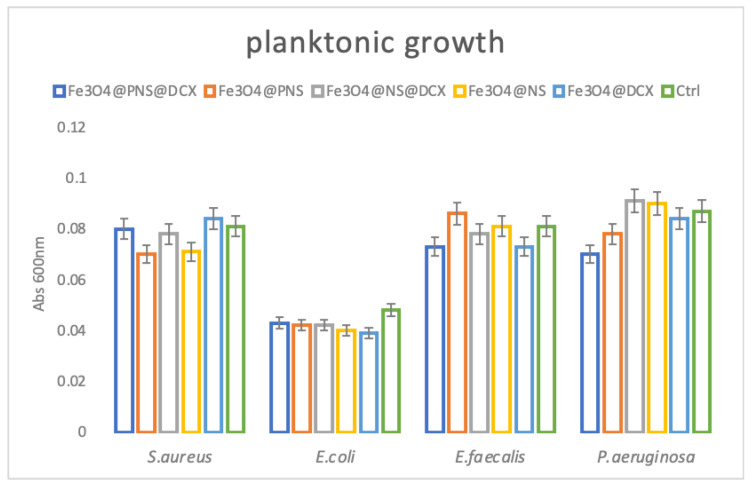
Evaluation of planktonic microbial cultures growth in the presence of control and nanocoatings for 24 h at 37 °C by graphical representation of average absorbance at 600 nm.

Regarding the antibacterial effect of the coatings, our study revealed that they have a limited effect in planktonic cultures, but are highly efficient in reducing microbial attachment and biofilm formation. These results were expected, as they prove that the bioactive compound (i.e., antibiotic, NS extracts) is embedded into the coating and that low amounts are released over time. Therefore, their maximum efficiency is reached during coating-microbial cell contact, and this modulates contact-dependent bacterial behaviours, such as attachment and biofilm development. Their planktonic growth impact can be observed in Figure 12 below. Here we can observe that planktonic cultured compounds are developing similarly in liquid media containing control and MAPLE processed coatings, suggesting that a low amount of the bioactive compounds which is released in the culturing media is not sufficient to significantly reduce microbial growth and development.

**Figure 13:**
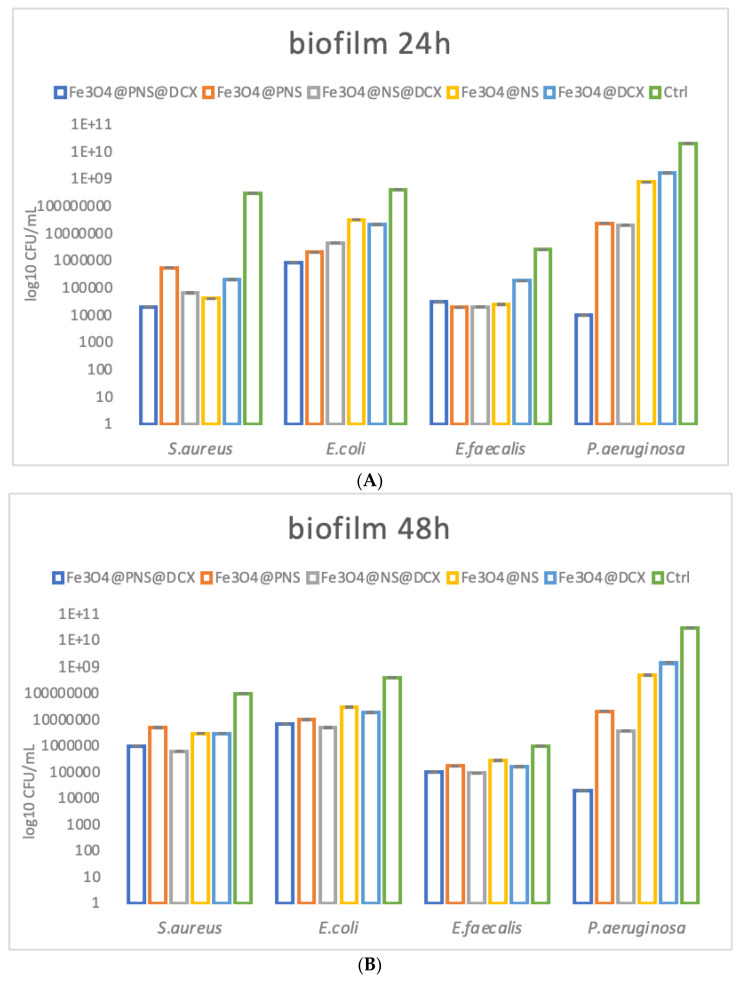

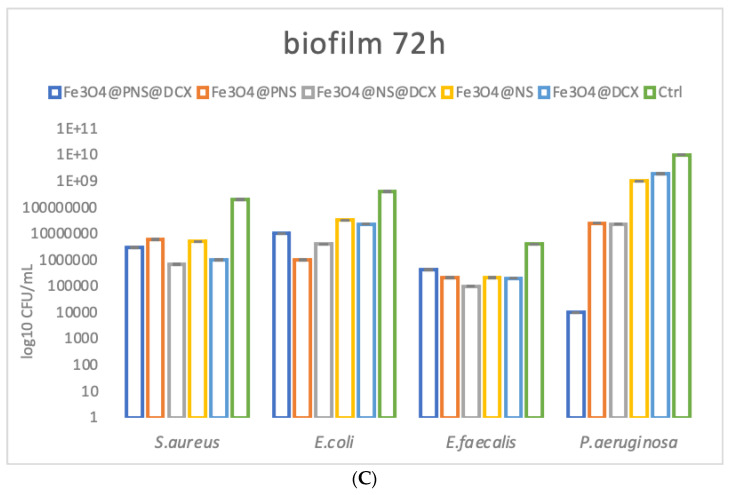
Graphical representation of log10 CFU/mL values obtained for the tested microbial strains, expressing biofilm embedded cells developed on control and coatings for different periods of time, after: 24 h (**A**), 48 h (**B**), and 72 h (**C**).

Biofilm growth represents one of the most common microbial strategies to develop difficult-to-treat infections. These multicellular bacterial communities could develop on various surfaces and materials, such as medical devices and coatings, and they represent one of the main causes of device failure and chronic wound infection. Bacteria existing in biofilms are resistant to almost any known antibiotic and to traditional biocide, which can be applied locally for wound management [79]. In this study we aimed to cover both planktonic and biofilm bacteria growth in order to evaluate the antimicrobial activity of the obtained MAPLE processed coatings. We have found that after 24 h, biofilms are considerably inhibited in the presence of the nanomodified coatings. Biofilm inhibition is related to the presence of bioactive agents within the coating. Therefore, samples containing Fe_3_O_4_@NS@DCX and Fe_3_O_4_@PNS@DCX showed the greatest antimicrobial effect (Figure 13A).

The inhibition of the biofilm is still active even after 48 h for all tested microbial species in the presence of coatings containing Fe_3_O_4_@NS@DCX and Fe_3_O_4_@PNS@DCX (Figure 13B).

Biofilm inhibition is diminished, after 72 h of contact, for *S. aureus*, *E. coli* and *E. faecalis* tested strains, but remains significant for *P. aeruginosa*, when coatings encoded Fe_3_O_4_@NS@DCX and Fe_3_O_4_@PNS@DCX are used. The highest biofilm development inhibition was observed for *P. aeruginosa*, the biofilms developed by this naturally resistant opportunistic pathogen being up to five-fold inhibited when an Fe_3_O_4_@PNS@DCX coating was used (Figure 13C).

These results suggest that the developed coatings may be very efficient in acting as a repelling dressing against the attachment of microbial pathogens, which could avoid wound colonization and subsequent biofilm development. This effect is most probably caused by the combined antimicrobial efficiency of the antibiotic (DCX) and NS extract, since both were previously reported as efficient antibacterial agents against wound pathogens [80,81,82]. 

#### 2.3.2. *In Vitro* Biocompatibility Assessment

**Figure 14:**
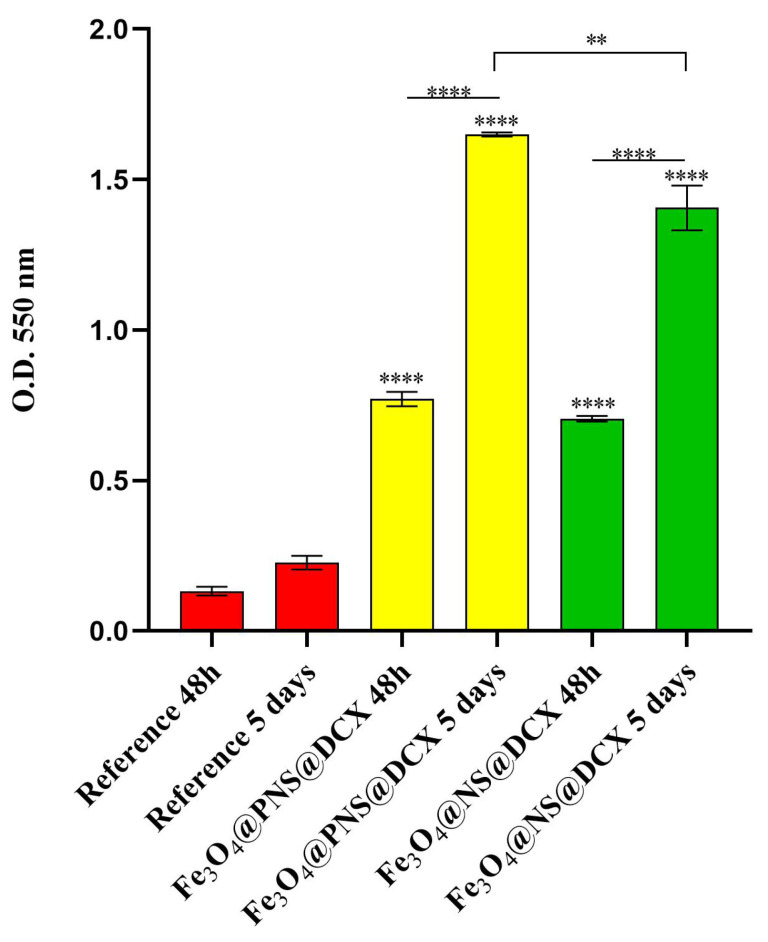
Graphical representation of human dermal fibroblast viability after 48 h and 5 days of contact with the wound dressings coated with Fe_3_O_4_@PNS@DCX and Fe_3_O_4_@NS@DCX. A pristine wound dressing was employed as the experimental control. Statistical significance: ** *p* < 0.01, **** *p* < 0.0001.

To evaluate the safety of the coatings, cell growth and proliferation were investigated using the MTT assay (Figure 14). In comparison with the reference sample, both coatings determined a statistically significant (**** *p* < 0.0001) increase in cell viability after 48h of culture, with no notable difference being observed between Fe_3_O_4_@PNS@DCX and Fe_3_O_4_@NS@DCX. After 5 days of culture, the cell viability maintained its upward trend on the coated samples as compared with the cell viability of dermal fibroblasts cultured on pristine wound dressings. However, at this time point, significant differences (** *p* < 0.01) between samples were observed, results that suggested that the form in which the natural compound was loaded into the coatings has a slight impact on cell viability, with Fe_3_O_4_@PNS@DCX revealing a higher ratio of viable cells than Fe_3_O_4_@NS@DCX. Moreover, the lack of dressing coating severely impacts the proliferative status of dermal fibroblasts, as highlighted by the modest increase of cell viability between 5 days and 48 h of culture on the reference samples. In the presence of the coated samples, CCD-1070Sk cells showed a statistically significant (**** *p* < 0.0001) increase in cell viability between 5 days and 48 h of culture, showing that the coating strategy stimulates and sustains human dermal fibroblast proliferation, regardless of the form (powder or oil) by which the natural compound is loaded into the coating structure.

**Figure 15:**
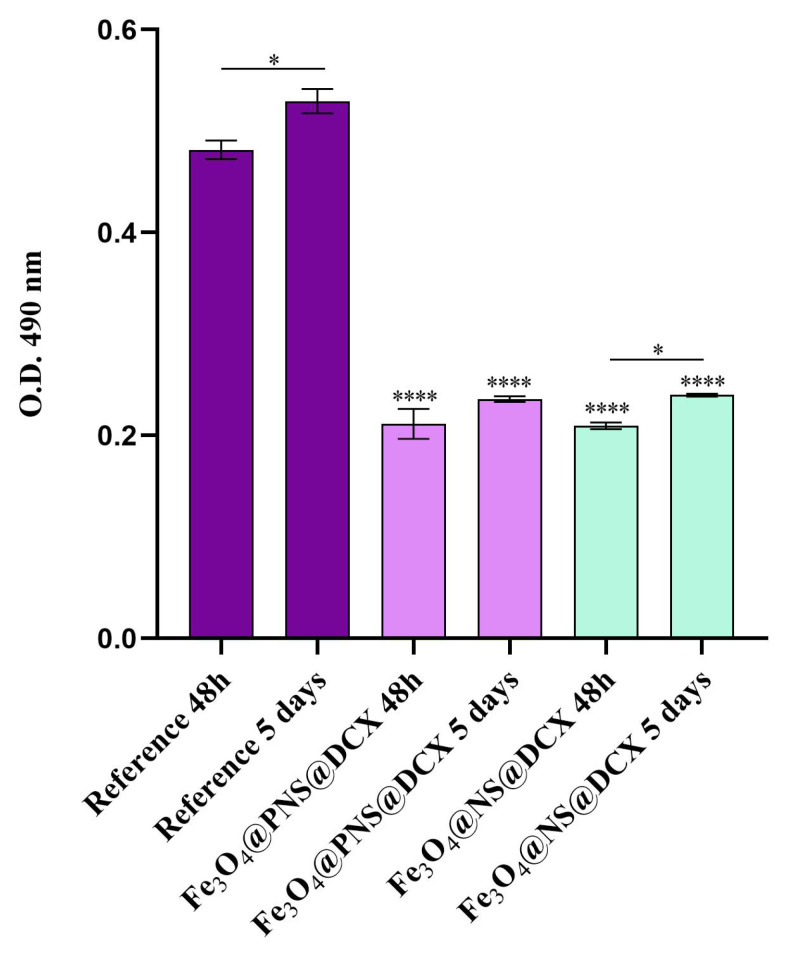
Cytotoxicity screening of Fe_3_O_4_@PNS@DCX and Fe_3_O_4_@NS@DCX-coated wound dressings after 48 h and 5 days of contact with the human dermal fibroblasts as revealed by the LDH assay. A pristine wound dressing was employed as the experimental control. Statistical significance: * *p* < 0.05, **** *p* < 0.0001.

The cytotoxicity screening results were in full agreement with the MTT assay (Figure 15). After 48 h of culture, the highest LDH levels were identified in culture medium samples harvested from the reference sample, with a statistically significant (**** *p* < 0.0001) decrease of the LDH levels in Fe_3_O_4_@PNS@DCX and Fe_3_O_4_@NS@DCX samples as compared with LDH levels registered in the reference samples. After 5 days of culture, the LDH release slightly increased for all tested samples, with no notable differences in the LDH enzyme level in the Fe_3_O_4_@PNS@DCX and Fe_3_O_4_@NS@DCX samples. In contrast, in the presence of the non-coated dressings, the LDH levels were significantly increased (* *p* < 0.5) after 5 days of culture as compared with levels registered after 48 h, showing that the pristine wound dressing exhibits significant higher cytotoxicity than the coated samples. 

**Figure 16:**
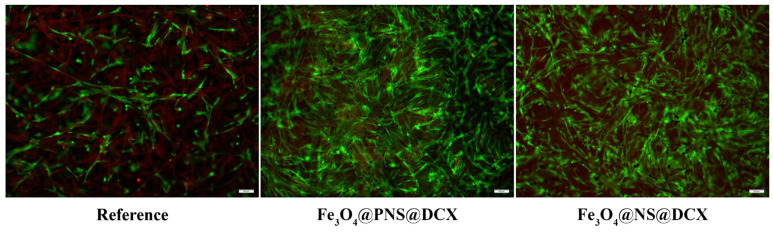
Fluorescence microscopy images showing live (green) and dead (red) human dermal fibroblasts after 5 days of contact with the reference sample.

Finally, to investigate the live and dead cell ratio on the wound dressing surfaces, as well as cell distribution on top of the samples, a Live/Dead assay was performed. A fluorescence microscopy (Figure 16) investigation of samples revealed the presence of dead cells only in the reference sample. In contact with the pristine wound dressing, a significantly lower ratio of live cells was observed on the sample compared to the ratio of live cells present on coated wound dressings. Regarding the Fe_3_O_4_@PNS@DCX and Fe_3_O_4_@NS@DCX samples, no obvious changes were observed between samples concerning the ratio of live cells identified. However, based on the coverage level of the sample surface, the Fe_3_O_4_@PNS@DCX coating was fully covered with cells that formed 3D complex intercellular networks in comparison with the Fe_3_O_4_@NS@DCX coating, where cells failed to cover the material surface completely, and more modest cellular networks were observed. In both samples, the human dermal fibroblast exhibited an overall elongated cell morphology characteristic of this particular cell line, showing that the coating structure does not impact cell morphology.

The obtained results showed that in the absence of a coating, the pristine wound dressing fails to sustain cell growth and proliferation, as revealed by the low survival rate of human dermal fibroblasts on the reference sample, as well as by the high cytotoxicity of this sample. Improving the wound dressing with Fe_3_O_4_@PNS@DCX and Fe_3_O_4_@NS@DCX triggered a positive shift of the investigated parameters related to human dermal fibroblasts. Therefore, tuning the wound dressing with either Fe_3_O_4_@PNS@DCX or Fe_3_O_4_@NS@DCX leads to obtaining a superior material that provides the appropriate conditions for human dermal fibroblast cell growth and development. Despite the excellent biocompatibility of both Fe_3_O_4_@PNS@DCX and Fe_3_O_4_@NS@DCX-coatings, a slight difference was noticed between samples that suggested that the human dermal fibroblasts presented a higher affinity for the Fe_3_O_4_@PNS@DCX coating.

## 3. Materials and Methods

### 3.1. Materials

The chemical substances used for the fabrication and functionalization of Fe_3_O_4_ NPs, i.e., ferrous sulfate (FeSO_4_), ferric chloride (FeCl_3_), acetic acid (CH₃COOH), ammonium hydroxide (NH_4_OH), doxycycline, and Dimethyl sulfoxide (DMSO) were purchased from Sigma-Aldrich Chemie GmbH (Merck KGaA, Darmstadt, Germany). *N. sativa*, in the form of powder (PNS) or as cold pressed and unrefined oil (NS), is commercially available. IR transparent silicon (Si) substrates (1 cm^2^ area) were provided by a local supplier. The cellulose discs (Φ = 12.7 mm) were purchased from ROTH (Karlsruhe, Germany). 

### 3.2. Synthesis Methods

#### 3.2.1. Synthesis of Magnetic NPs Functionalized with DCX 

Fe_3_O_4_ NPs were obtained by the co-precipitation method from Fe^2+^ and Fe^3+^ (1:2 molar ration) according to the literature [83,84]. The concentration of *N. sativa* (powder/oil) in aqueous NH_4_OH solution was 0.25%. The product was repeatedly washed with methanol and subsequently dried in an oven at 40 °C until reaching a constant weight [85]. The Fe_3_O_4_/*N. sativa* nano-support and the DCX antibiotic to be adsorbed were mixed at room temperature in the presence of 1 mL of chloroform until the latter has been completely evaporated. The amount of the DCX adsorbed on the nano-support was selected to be 3 mg. 

#### 3.2.2. MAPLE Target Preparation

A DMSO of 2.5% (*w*/*v*) Fe_3_O_4_@NS or Fe_3_O_4_@PNS and Fe_3_O_4_@NS/PNS@DCX nanoparticles was prepared. All MAPLE target solutions were poured into a pre-cooled target holder at 77 K and subsequently immersed in liquid nitrogen for 30 min.

#### 3.2.3. Magnetite-Based Coatings Synthesis by MAPLE

Targets used in MAPLE experiments were Fe_3_O_4_@NS, Fe_3_O_4_@PNS, Fe_3_O_4_@NS@DCX and Fe_3_O_4_@PNS@DCX. MAPLE depositions were performed using a KrF* (λ = 248 nm and τ_FWHM_ = 25 ns) laser source COMPexPro 205 model (Lambda Physics-Coherent) operating at the repetition rate of 15 Hz. The energy of a laser pulse was ~420 mJ concentrated in a spot with an area of ~29 mm^2^. The target and the substrate holder were in a plan-parallel configuration at a separation distance of 4 cm. The number of subsequent laser pulses for the deposition of one single coating was situated in the (80,000–110,000) interval. The experiments were performed in a stainless steel vacuum chamber under an ambient pressure of approximately 10^−4^ mbar. 

**Figure 17:**
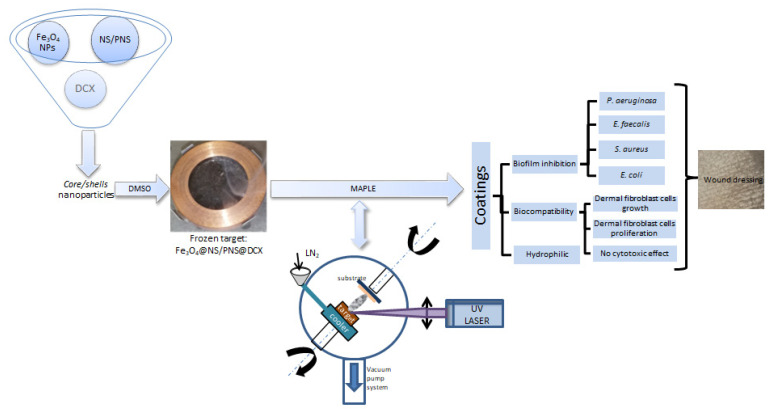
Schematic representation of the whole process of obtaining coatings based on Fe_3_O_4_ NPs functionalised with *N. sativa* and DCX.

For easier understanding, the whole process of obtaining coatings based on Fe_3_O_4_ NPs functionalised with *N. sativa* and DCX is schematically presented in Figure 17.

### 3.3. Physicochemical Characterization

#### 3.3.1. X-ray Diffraction (XRD)

The crystalline nature of both nanopowder and coatings was investigated by XRD using an Empyrean X-ray diffractometer from PANalytical (Almelo, The Netherlands,), with CuKα radiation (λ = 1.54 Å). The diffractometer was operated at a generator power of 45 kV and 40 mA in a parallel beam geometry using a parabolic mirror for X-radiation Cu Kα with a 1/8° slit in front of the incident beam and a parallel beam collimator of 0.27° in front of the X-ray detector for the diffracted beam. Diffraction figures were analysed using the Panalytical HighScore Plus™ software package and the International Center of Diffraction Data (ICDD) 2020 database.

#### 3.3.2. Transmission Electron Microscopy (TEM) 

For TEM investigations, a JEOL JEM ARM 200 F electron microscope operated at 200 keV was used. Samples for TEM were prepared by suspending them in ethanol and transferring them to a copper grid coated with an amorphous carbon support. The particles sizes were established from the measurement of ~100 particles for each sample.

#### 3.3.3. Thermogravimetric Analysis with Differential Scanning Calorimetry (TGA-DSC)

For TGA-DSC analysis, a small quantity of powder was placed in an open alumina crucible and heated from room temperature to 900 °C at a heating rate of 10 °C/min under a dynamic air atmosphere. An empty alumina crucible was used as a reference. TGA-DSC analyses were performed using a Netzsch STA 449C Jupiter (NETZSCH-Gerätebau GmbH, Selb, Germany). 

#### 3.3.4. Scanning Electron Microscopy (SEM)

The morphology and dimensions of nanostructures were investigated by scanning electron microscopy (SEM) using a FEI InspectS microscope which operates under high and low vacuum conditions, with accelerating voltages between 200 V and 30 kV, and a maximum 50 nm resolution.

#### 3.3.5. Fourier-Transform Infrared Spectroscopy (FT-IR)

For FT-IR investigations we used a Nicolet 6700 FT-IR spectrometer (Thermo Fisher Scientific, Waltham, MA, USA). As a result, 32 scans of each sample were realized at room temperature, in a frequency range of 4000–1000 cm^−1^, and a 4 cm^−1^ spectral resolution. The acquired information was recorded by connecting the spectrometer to a unity of data processing using the Omnic Picta 8.2 software (Thermo Fischer Scientific).

#### 3.3.6. Drug Release

In order to simulate the interaction of wound dressings with human body tissues and to study the phenomena occurring at the dressing-tissue interface as a result of the interaction with physiological fluids, the coated cellulose discs were immersed in 2 mL of simulated body fluid (SBF) and investigated after 8 h. The SBF, having an ionic composition identical to that of blood plasma, was prepared according to the Kokubo formula [86]. Samples immersed in SBF were maintained at 37 °C (human body temperature). The SBF containing the products released by the samples for 8h was analysed by UV-Vis absorption spectroscopy with a ThermoFisher Scientific Evolution 220 spectrophotometer. The analysis range used was 190–1200 nm in absorbance mode. All measurements were performed in triplicate according to ISO/FDIS 23317:2007(E).

### 3.4. Biological Characterization

#### 3.4.1. Planktonic Growth in Nutritive Broth

The effect of the MAPLE coatings on the growth of microorganisms in planktonic cultures was evaluated. The MAPLE coatings were sterilized by UV exposure for 20 min before analysis. A piece of the sterile coatings was individually deposited in a well of a sterile 24-well plate. Next, 1 mL of nutritive broth and subsequently 10 μL of 0.5 McFarland density microbial suspensions prepared in PBS (phosphate buffered saline) were added over the coatings. The prepared plates were incubated at 37 °C for 24 h. A total of 150 μL of the obtained microbial culture (planktonic cells) was transferred to 96 well plates and the turbidity of the microbial cultures (absorbance, Abs 600 nm) was measured spectrophotometrically [87].

#### 3.4.2. Bacterial Strains

The antibacterial evaluation of the nanomodified wound dressings was assessed *in vitro* against four microbial strains, known for their ability to produce biofilms and wound infections, namely two Gram positive (*S. aureus* ATCC 23235 and *E. feacalis* ATCC 29212) and two Gram negative (*E. coli* ATCC 25922 and *P. aeruginosa* ATCC 27853) bacteria.

#### 3.4.3. Monospecific Biofilm Development 

In this assay, we have evaluated the short-term and long-term antimicrobial efficiency against monospecific biofilms, following a protocol described by [87]. The antibiofilm efficiency was established by evaluating the bacterial biofilm development in the presence of control and MAPLE modified coatings. The obtained thin coatings were placed in sterile 24-well plates in 1 mL of liquid media (nutritive broth), followed by the inoculation of 10 μL of microbial suspension of 0.5 McFarland standard density from each bacterial strain. The samples were then incubated for 24 h at 37 °C. Afterwards, the culture media was removed and the samples were washed with 1 mL sterile PBS. This washing step aims to remove the unattached bacteria. The samples were then transferred to sterile 24-well plates containing fresh media and incubated for 24, 48, and 72 h, respectively, at 37 °C to allow the growth and biofilm formation of the attached bacteria. The wound dressing samples were then gently washed with sterile phosphate-buffered saline and further placed in 1.5 mL centrifuge tubes containing 1000 μL of PBS. The obtained specimens were vortexed for 30 s and subsequently subjected to ultrasounds for 10 s to detach the biofilm cells and obtain microbial suspensions of cells which were previously embedded into biofilms. Serial ten-fold dilutions were performed from the obtained suspensions and inoculated on nutrient agar for viable cell count assays.

#### 3.4.4. *In Vitro* Biocompatibility Assessment

The CCD-1070Sk human dermal fibroblasts cell line (ATCC^®^ CRL-2091™, ATCC, American Type Collection, Manassas, VA, USA) was employed as an *in vitro* cellular model for assessing the biocompatibility of the improved wound dressings. Cells were maintained in Dulbecco’s Modified Eagle’s Medium (DMEM, Sigma/Merck, Steinheim, Germany), supplemented with 10% fetal bovine serum (FBS, Gibco, Thermo Fischer Scientific, Waltham, MA, USA) and a 1% penicillin-streptomycin mix (Sigma/Merck), under a humid atmosphere with 5% CO_2_ at 37 °C. Before the biocompatibility assessment, all the experimental samples were sterilized for 20 min by UV light exposure and transferred aseptically in 24-well culture plates. A pristine wound dressing was used as a reference for all cell culture-based assays and was processed identically to the coated samples. Cells were seeded on simple and coated-wound dressings in a droplet at an initial density of 104 cells/cm^2^. After 2 h, the complete culture medium was added in all wells containing samples and further incubated for 5 days in standard cell culture conditions.

The MTT assay (3-[4,5-dimethylthiazol-2-yl]-2,5 diphenyl tetrazolium bromide, Sigma/Merck) was performed to investigate cell viability and proliferation potential after 48 h and 5 days of fibroblasts’ contact with the simple and coated-wound dressings based on the metabolic activity of healthy cells. The culture medium was discarded and replaced with a fresh MTT solution (1 mg/mL) prepared in serum-free DMEM. After 4 h of incubation with the MTT solution (5% CO_2_, 37 °C), the resulting formazan crystals were solubilized using DMSO and the optical densities (OD) of the resulting solutions were measured at 550 nm using the multimodal FlexStationIII reader (Molecular Devices, San Jose, CA, USA). 

The cytotoxic potential of the simple and coated wound dressings was investigated by measuring the levels of lactate dehydrogenase (LDH) released in the culture medium by damaged fibroblasts. For this, medium samples were harvested 48 h and 5 days after experiment initiation and mixed with the components of the “*In vitro* toxicology assay kit lactate dehydrogenase based TOX—7” kit (Sigma/Merck) according to the manufacturer’s recommendations. After 30 min of incubation at RT in the dark, the absorbance of the resulting solutions was read at 490 nm using the multimodal reader FlexStation III to determine cell membrane damage and cell death induced by sample contact.

All of the experiments were performed in triplicate and all the statistical data are presented as mean values ± standard deviation of three independent experiments. For statistical analyses, GraphPad Prism software (San Diego, CA, USA) was employed, using one-way analyses of variance (ANOVA), with Bonferroni’s multiple comparisons post-test used to identify which groups were different. Results with *p* < 0.05 were considered statistically significant.

To simultaneously highlight live and dead cells on the samples’ surface and reveal the overall cellular distribution after 5 days of culture, a Live/Dead kit (Invitrogen, Life Technologies, Foster City, CA, USA) was used. Briefly, as recommended by the manufacturer’s instructions, a mix of calceinAM and ethidium bromide was prepared in PBS (Sigma/Merck). After discarding the culture medium, samples were immersed in the prepared solution for 20 min in the dark at RT and consequently analyzed using the Olympus IX73 fluorescence microscope and CellSense F software version 1.11. 

## 4. Conclusions

The Fe_3_O_4_@PNS@DCX coating was amorphous in comparison to Fe_3_O_4_@NS@DCX and Fe_3_O_4_@DCX, respectively. In contrast, an XRR analysis showed that the amorphous coating is much denser than the crystalline ones. An FTIR analysis revealed that the laser transfer did not affect the chemical integrity of the material for all coatings containing DCX.

Following the evaluation of the release process of the active substance, it was observed that a significant amount of the drug released during the 8 h of testing destroys the adhered microbial strains, while a smaller amount of the drug released in the following days prevents the formation of the bacterial biofilm that can cause infections.

The most effective antimicrobial effect was observed in the case of dressings coated with Fe_3_O_4_@PNS@DCX, this being highlighted both in terms of the growth and multiplication of microorganisms, as well as in their ability to develop monospecific biofilms.

The Fe_3_O_4_@PNS@DCX and Fe_3_O_4_@NS@DCX coatings are biocompatible, being able to support the viability and proliferation of dermal fibroblasts, in addition to being free of cytotoxic effects on the chosen *in vitro* cell model.

## Data Availability

Not applicable.
